# Genetic Screening of the Usher Syndrome in Cuba

**DOI:** 10.3389/fgene.2019.00501

**Published:** 2019-05-22

**Authors:** Elayne E. Santana, Carla Fuster-García, Elena Aller, Teresa Jaijo, Belén García-Bohórquez, Gema García-García, José M. Millán, Araceli Lantigua

**Affiliations:** ^1^Centro Provincial de Genética, Universidad de Ciencias Médicas de Holguín, Holguín, Cuba; ^2^Health Research Institute La Fe, University Hospital La Fe, Valencia, Spain; ^3^Centro de Investigación Biomédica en Red de Enfermedades Raras (CIBERER-ISCIII), Madrid, Spain; ^4^Centro Nacional de Genética Médica, Havana, Cuba

**Keywords:** retinitis pigmentosa, sensorineural hearing loss, Usher syndrome, deaf-blindness, molecular genetics

## Abstract

**Background:**

Usher syndrome (USH) is a recessive inherited disease characterized by sensorineural hearing loss, retinitis pigmentosa, and sometimes, vestibular dysfunction. Although the molecular epidemiology of Usher syndrome has been well studied in Europe and United States, there is a lack of studies in other regions like Africa or Central and South America.

**Methods:**

We designed a NGS panel that included the 10 USH causative genes (*MYO7A*, *USH1C*, *CDH23*, *PCDH15*, *USH1G*, *CIB2*, *USH2A*, *ADGRV1*, *WHRN*, and *CLRN1*), four USH associated genes (*HARS*, *PDZD7*, *CEP250*, and *C2orf71*), and the region comprising the deep-intronic c.7595-2144A>G mutation in *USH2A*.

**Results:**

NGS sequencing was performed in 11 USH patients from Cuba. All the cases were solved. We found the responsible mutations in the *USH2A*, *ADGRV1*, *CDH23*, *PCDH15*, and *CLRN1* genes. Four mutations have not been previously reported. Two mutations are recurrent in this study: c.619C>T (p.Arg207^∗^) in *CLRN1*, previously reported in two unrelated Spanish families of Basque origin, and c.4488G>C (p.Gln1496His) in *CDH23*, first described in a large Cuban family. Additionally, c.4488G>C has been reported two more times in the literature in two unrelated families of Spanish origin.

**Conclusion:**

Although the sample size is very small, it is tempting to speculate that the gene frequencies in Cuba are distinct from other populations mainly due to an “island effect” and genetic drift. The two recurrent mutations appear to be of Spanish origin. Further studies with a larger cohort are needed to elucidate the real genetic landscape of Usher syndrome in the Cuban population.

## Introduction

Usher syndrome (USH, OMIM 276900, OMIM 276905, OMIM 605472, ORPHA: 886) is the most prevalent deaf-blindness of genetic origin. It is a recessive inherited disease characterized by sensorineural hearing loss (HL), visual loss due to retinitis pigmentosa (RP), and, in some cases, vestibular dysfunction. Prevalence estimates range from 3.2 to 6.2/100,000 (Espinós et al., 1998; Keats and Corey, 1999).

Patients with USH are classified into three clinical subtypes (USH1, USH2, or USH3), based on the severity and progression of hearing impairment and the presence or absence of vestibular dysfunction. Usher syndrome type I (USH1) is the most severe type, characterized by severe to profound congenital sensorineural hearing loss, vestibular dysfunction, and prepubertal onset of RP eventually leading to legal blindness. USH2 is characterized by moderate to severe hearing impairment, normal vestibular function and later onset of retinal degeneration. USH3 displays progressive hearing loss, RP and variable vestibular phenotype (Saihan et al., 2009; Millán et al., 2010).

Currently, up to 13 genes have been associated with Usher syndrome: *MYO7A*, *USH1C*, *CDH23*, *PCDH15*, *USH1G*, and *CIB2* are responsible for USH1, although the role of *CIB2* in the Usher syndrome has recently been put on doubt (Booth et al., 2018). *USH2A*, *ADGRV1*, and *WHRN* are the three genes responsible for USH2, and the *CLRN1* gene is the only one associated with USH3 cases to date. Besides, *PDZD7* has been reported to behave as a modifier of the retinal phenotype in conjunction with *USH2A*, and a contributor to digenic inheritance with *ADGRV1* (Ebermann et al., 2010). In addition, *HARS* was postulated as a novel causative gene of USH3, based on a mutation found in two patients (Puffenberger et al., 2012). Finally, mutations in *CEP250* have been reported to cause cone-rod dystrophy, isolated RP and atypical forms of USH, characterized by early onset hearing loss and mild RP (Khateb et al., 2014; Fuster-García et al., 2018; Kubota et al., 2018).

In the last years, next generation sequencing (NGS) techniques have revolutionized the world of the molecular genetic diagnosis, allowing the whole genome, whole exome and targeted gene sequencing more feasible, and making easier, rapid and cost-effective the identification of disease genes and the underlying mutations. It has been especially useful in genetically heterogeneous diseases, such as hearing loss or retinal dystrophies (Choi et al., 2013; Fu et al., 2013; Mutai et al., 2013; Glöckle et al., 2014). We previously developed a targeted next generation sequencing method for Usher syndrome that proved to be highly efficient (Aparisi et al., 2014; Fuster-García et al., 2018).

Although the molecular epidemiology of the Usher syndrome and the distribution of mutations causing the disease among these genes has been well studied in Europe and United States, there is a lack of studies in other regions like Africa or Central and South America.

Here, we show for the first time a molecular landscape of the Usher syndrome in Cuba, and we provide as well a clinical description of all the cases.

## Materials and Methods

### Patients

**FIGURE 1:**
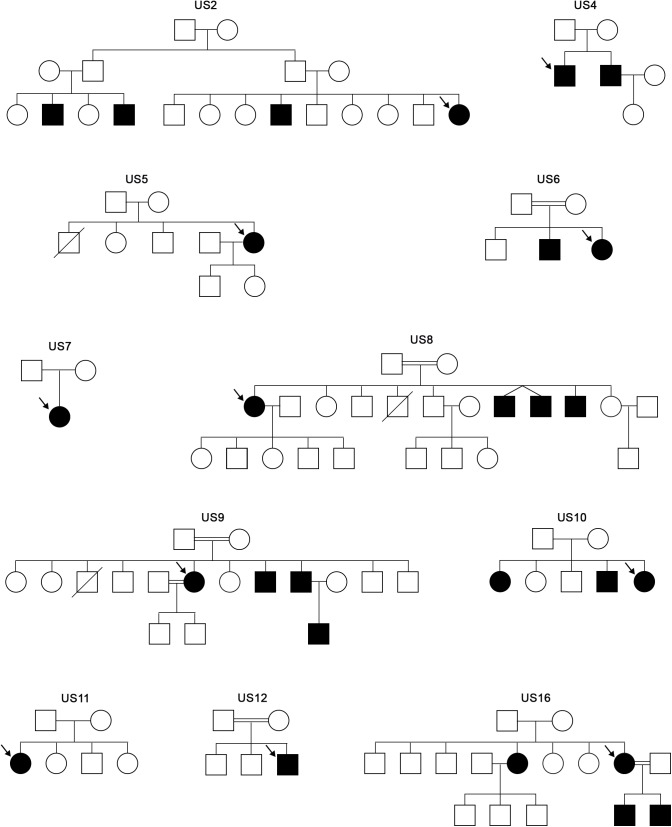
Family trees of the Usher families analyzed in this study. Arrows indicate the proband that was studied through the USH targeted panel.

A descriptive cross-sectional study was carried out in a series of 11 families from Holguin (Cuba) with patients diagnosed clinically as Usher syndrome. All the 11 patients were Caucasian. The family trees of the families are shown in Figure 1.

The variables collected in this study were: age, sex, ethnicity, birthplace of the patients and their ancestors, consanguinity, age of onset HL and at diagnosis, HL degree, age of the first symptoms of RP and current clinical stage, and vestibular function. The institutional board of both the Ethics Committee of the University Hospital La Fe and the University of Holguín approved the study, according to the tenets of the Declaration of Helsinki and reviews. A survey assessed by the researchers was used in compliance after signing informed consent.

Ophthalmological examination included visual acuity, funduscopy, visual field test with Goldmann perimetry, and electroretinogram (ERG). The Audiological examination consisted of the vestibular function study through the caloric test and study of brainstem auditory evoked potentials (BAEP).

Hearing loss evaluation was carried out using a radio audiometer MA31 (Grosses Klinisches Audiometer, Germany) in the Hospital “Vladimir Ilich Lenin.” The BAEPs were obtained in response to the monaural stimulation through TDH-39 hearing aids, with condensation clicks with a duration of 100 μsec and an intensity of 95 dB pSPL. The hearing loss of each affected individual was quantified by performing a complete tonal audiometry. Hearing loss was classified as: Mild (20–40 dB), moderate (40–70 dB), severe (70–90 dB), or profound (more than 90 dB).

Peripheral blood was obtained and DNA was extracted in the National Center for Medical Genetics in Havana, and sent to the University Hospital La Fe in Valencia (Spain).

### Targeted Exome Sequencing Design

We designed a customized AmpliSeq panel using Ion AmpliSeq Designer tool from Thermo Fisher Scientific^1^ to generate the targeted library composed of all exons contemplated in all isoforms with 10 bp padding of the flanking intron regions, and the additional locus comprising the c.7595-2144A>G intronic mutation (Vaché et al., 2012). These target regions were covered by 810 amplicons of 125–175 bp length range, computing a total panel size of 147.95 kb. The designed panel (Table 1) included 14 genes, 10 USH causative genes (*MYO7A*, *USH1C*, *CDH23*, *PCDH15*, *USH1G*, *CIB2*, *USH2A*, *ADGRV1*, *WHRN*, and *CLRN1*) and four USH associated genes (*HARS*, *PDZD7*, *CEP250*, and *C2orf71*).

**Table 1:** Details of the target region studied in this study.

Chr	Gene/locus	Coding exons	Size (bp)	Number of amplicons	Design coverage
5	*ADGRV1*	90	20721	181	99.4%
1	*USH2A*	72	17043	134	98.9%
10	*CDH23*	73	11849	120	99.5%
10	*PCDH15*	43	8284	67	98.2%
20	*CEP250*	32	7969	58	100%
11	*MYO7A*	51	7642	88	98.6%
2	*C2orf71*	2	3907	23	99.6%
10	*PDZD7*	17	3474	31	97.5%
11	*USH1C*	29	3334	38	94.2%
9	*WHRN*	14	2964	26	100%
5	*HARS*	15	1790	14	100%
17	*USH1G*	4	1446	12	100%
3	*CLRN1*	9	1051	9	100%
15	*CIB2*	7	684	8	95%
1	^∗^chr1: 216064460-216064620	–	160	1	100%

*Chr, chromosome number. ^∗^Region of the USH2A PE (Pseudo-exon 40) where mutation c.7595-2144A>G is located. Targets are arranged according to the size of the covered region.*

### Sequence Enrichment and Next Generation Sequencing

The amplification of the targets was performed according to the Ion AmpliSeq Library Kit 2.0 protocol (Thermo Fisher Scientific, Inc.) for Ion Torrent sequencing. The sequencing was carried out with a theoretical minimum coverage of 500× either on the *PGM* or *Proton* system.

### Variant Filtering and Analysis

The resulting sequencing data were analyzed with the Ion Reporter Software tool ^2^ in regards to the human assembly GRCh37 (also known as hg19). The annotated variants were filtered according to a Minor Allele Frequency (MAF) value ≤0.01, their annotation in the dbSNP^3^, their description in the Usher syndrome mutation database^4^ and the mutation type. Those disease-causing and suspected-to-be pathogenic variants were validated through conventional Sanger sequencing. For this, each DNA *locus* comprising a selected mutation was amplified by PCR with specific primers, and both forward and reverse strands were sequenced using the Big Dye 3.1 Terminator Sequencing Kit (Thermo Fisher Scientific, Inc.) after enzymatic PCR clean up with illustra ExoProStar 1-Step (GE Healthcare Life Sciences). The purified sequence products were analyzed on a 3500xL ABI instrument (Applied Biosystems by Thermo Fisher Scientific, Inc.).

The novel variants found in the cohort of probands were categorized based on the guidelines of the *clinical and molecular genetics society*^5^ and the Unknown Variants classification system (see text footnote 4) as pathogenic, probably pathogenic (UV4), possibly pathogenic (UV3), possibly non-pathogenic (UV2), and neutral (UV1), according to the type of mutation, bioinformatic predictions and segregation analysis. The four novel mutations were frameshift or nonsense mutations. Hence, they were automatically stated as pathogenic variants.

The annotation of the variants was performed according to following isoform reference sequences for each gene: *MYO7A* (NM_000260.3), *USH1C* (NM_153676), *CDH23* (NM_022124.5), *PCDH15* (NM_033056.3), *USH1G* (NM_173477), *CIB2* (NM_006383.2), *USH2A* (NM_206933), *ADGRV1* (NM_032119.3), *WHRN* (NM_015404), *CLRN1* (NM_174878), *HARS* (NM_002109), *PDZD7* (NM_001195263.1), *CEP250* (NM_007186.4), and *C2orf71* (NM_001029883.2).

### MLPA Complementary Analysis

In order to ascertain if homozygous mutations could truly be masked cases of a large deletion comprising a heterozygous variant, we performed pertinent multiplex Multiplex ligation-dependent probe amplification (MLPA; MRC-Holland) analysis for the only USH genes available, *USH2A* and *PCDH15*.

**Table 2:** Genetic findings of the patients screened in this study, mutations, their effect on the protein, genes mutated, and nature of the mutations.

Patient	Diagnosis	Mutations	Effect on protein	Gene	Type of mutation	References
US-2	USH2	c.15448_15449delCT	p.Leu5150Hisfs^∗^6	ADGRV1	Frameshift	This study
		c.15448_15449delCT	p.Leu5150Hisfs^∗^6			
US-4	USH1	c.4488G>C	p.Gln1496His	CDH23	Splice site	Bolz et al., 2001
		c.7730_7734delTCAGT	p.Phe2577Serfs^∗^28		Frameshift	This study
US-5	USH1	c.4488G>C	p.Gln1496His	CDH23	Splice site	Bolz et al., 2001
		c.4488G>C	p.Gln1496His			
US-6	USH1	c.4488G>C	p.Gln1496His	CDH23	Splice site	Bolz et al., 2001
		c.4488G>C	p.Gln1496His			
US-7	USH1	c.4488G>C	p.Gln1496His	CDH23	Splice site	Bolz et al., 2001
		c.1624G>T	p.Glu542^∗^		Nonsense	This study
US-8	USH?	c.619C>T	p.Arg207^∗^	CLRN1	Nonsense	García-García et al., 2012
		c.619C>T	p.Arg207^∗^			
US-9	USH2	c.2299delG	p.Glu767Serfs^∗^21	USH2A	Frameshift	Liu et al., 1999
		c.2299delG	p.Glu767Serfs^∗^21			
US-10	USH2	c.3661C>T	p.Gln1221^∗^	PCDH15	Nonsense	This study
		c.3661C>T	p.Gln1221^∗^			
US-11	USH1	c.4488G>C	p.Gln1496His	CDH23	Splice site	Bolz et al., 2001
		c.4488G>C	p.Gln1496His			
US-12	USH?	c.619C>T	p.Arg207^∗^	CLRN1	Nonsense	García-García et al., 2012
		c.619C>T	p.Arg207^∗^			
US-16	USH2	c.1841-2 A>G	p.Gly614Aspfs^∗^6	USH2A	Splice site	Jaijo et al., 2010
		c.1841-2 A>G	p.Gly614Aspfs^∗^6			

## Results

Eleven index cases diagnosed of Usher syndrome from the province of Holguín, Cuba, were screened for mutations in the USH-associated genes of our home-designed panel.

Details of the genes, number of amplicons or coverage are described in Table 1.

Five cases were diagnosed of USH1, whereas four cases were USH2, and two cases were difficult to classify clinically. All the eleven cases were solved and the specific causative mutations can be found in Table 2.

Six families were consanguineous (54.5%) and another two were probably consanguineous (18.2%), since the parents come from the same small village. In total, the consanguinity or probable consanguinity in the cohort is over 70%.

Among the USH1 cohort, two pathogenic mutations were found in *CDH23* (US-4, US-5, US-6, US-7, and US-11). In the USH2 cohort, two pathogenic mutations were found in *ADGRV1* (US-2) and *USH2A* (US-16 and US-9), and *PCDH15* (US-10). Regarding the unclassified cases, two mutations were found in *CLRN1* (US-8 and US-12). Patient US-2, who carried the mutation c.15448_15449delCT in homozygosis in the *ADGRV1* gene, carried the additional c.3242G>A (p.Arg1081Gln) missense mutation in *CDH23* in heterozygous state, which is predicted to probably damaging according to PolyPhen-2 and benign as SIFT and PROVEAN.

Four mutations are reported in this study for the first time, namely c.15448_15449delCT (p.Leu5150Hisfs^∗^6) in *ADGRV1*, c.7730_7734delTCAGT (p.Phe2577Serfs^∗^28) in *CDH23*, c.1624G>T (p.Glu542^∗^) in *CDH23*, and c.3661C>T (p.Gln1221^∗^) in *PCDH15*.

Two mutations have been found in several USH alleles in this study. The p.Arg207^∗^ mutation in *CLRN1* was found in homozygous state in two different families, both of them consanguineous. That means 18.2% of the total mutated alleles and 40% among the non-USH1 mutated alleles. Among the USH1 cases, p.Gln1496His accounted for 80% of the USH1 alleles (eight out of 10) and 36.4% of the total USH alleles. All the USH1 patients bear mutations in *CDH23*.

**FIGURE 2:**
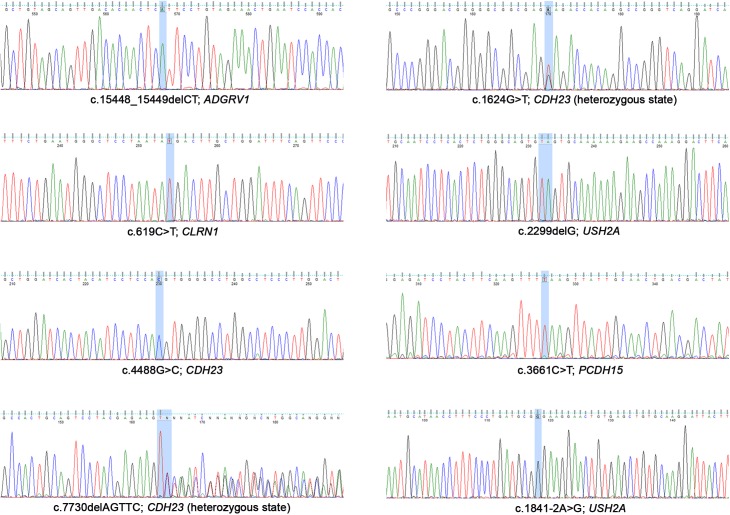
Sanger electropherograms of the mutations detected in this study.

The sequences of each mutation are shown in Figure 2.

MLPA assays in the patients US-9, US-10, and US-16, with homozygous mutations in either *USH2A* or *PCDH15*, revealed no copy number variations.

### Clinical Description

The clinical features of the 11 index patients are shown in Table 3.

#### Mutation: c.15448_15449delCT (p.Leu5150Hisfs^∗^6) in *ADGRV1*

Proband of family US-2: The subject comes from a non-consanguineous family (father from Mexico and mother from Cuba) and displays a typical USH2 phenotype. She presented with a postlingual moderate non-progressive HL, no vestibular dysfunction and postpubertal onset of RP. This patient carries the mutation p.Leu5150Hisfs^∗^6 in *ADGRV1* in homozygosis.

#### Mutation: c.619C>T (p.Arg207^∗^) in *CLRN1*

Proband of family US-8: Patient coming from a consanguineous family, harboring the mutation p.Arg207^∗^ in *CLRN1* in homozygous state. She has a postlingual moderate HL with a progression in the last 10 years. This subject is 81 years old and the progression of the HL may be due to age-related hearing impairment. She noticed nyctalopia at 8 years old and the visual field was much reduced by the age of diagnosis. She did not report any balance problems.

Proband of family US-12: Patient carries the p.Arg207^∗^ mutation in *CLRN1* in homozygous state. The family is also consanguineous, since the parents are second cousins. HL is postlingual and severe. RP signs were similar than those of US-8, yet with a later age of onset of symptoms and reduced visual field at age 24. ERG is abolished for this subject. In addition, the delayed walking onset and the reported difficulties in holding the head up as a baby suggest balance dysfunction.

#### Mutation: c.1841-2 A>G (p.Gly614Aspfs^∗^6) in *USH2A*

Proband of family US-16: The subject has a typical *USH2A* phenotype with a moderate postlingual non-progressive HL and typical RP of onset in the puberty.

#### Mutation: c.2299delG (p.Glu767Serfs^∗^21) in *USH2A*

Proband of family US-9: The patient harbors the most common mutation in *USH2A* patients of European origin, namely the c2299delG in *USH2A*. She displays a typical USH2 phenotype milder that US-16 with a mild postlingual HL and later onset of RP symptoms.

#### Mutation: c.3661C>T (p.Gln1221^∗^) in *PCDH15*

Proband of family US-10: This patient carries the p.Gln1221^∗^ mutation in *PCDH15* in homozygous state. *PCDH15* is associated to USH1 phenotype, however, this subject displayed postlingual moderate HL, normal vestibular function and relatively late-onset of RP.

#### Mutation: c.7730_7734delTCAGT (p.Phe2577Serfs^∗^28) in *CDH23*

Proband of family US-4: The patient is a compound heterozygote for the *CDH23* mutations p.Phe2577Serfs^∗^28 and p.Gln1496His. He displays a typical USH1 phenotype with a prelingual, severe hearing loss RP onset al puberty and vestibular dysfunction.

#### Mutation: c.1624G>T (p.Glu542^∗^) in *CDH23*

Proband of family US-7: Compound heterozygote for the *CDH23* mutations p.Glu542^∗^ and p.Gln1496His. Symptoms are distinctive of typical USH1 phenotype with a prelingual severe HL, early onset of RP and vestibular dysfunction.

#### Mutation: c.4488G>C (p.Gln1496His) in *CDH23*

Besides the compound heterozygotes US-4 and US-7, that carry p.Gln1496His together with other *CDH23* mutations, three more patients carry the mutation in homozygous state, namely those from families US-5, US-6, and US-11. All of them displayed a typical USH1 phenotype.

## Discussion

In this work, we report the first study in a cohort of Usher syndrome patients from Cuba. We found a total of eight mutations in 11 cases, four of which are novel (p.Leu5150Hisfs^∗^6 in *ADGRV1*, p.Phe2577Serfs^∗^28 and p.Glu542^∗^ in *CDH23*, and p.Gln1221^∗^ in *PCDH15*).

**Table 3:** Clinical features of the Cuban patients.

Family	Consanguinity	Case (age)	Type	Audiological findings	Vestibular function	Ophthalmological findings			
						NB	RP Dx	Clinical findings	ERG
US-2	No	III:2 (76)	USH2	Moderate SNHL; postlingual (adolescence); non-progressive	Normal	8 yo	14 yo	VF constriction (tunnel vision); pallor of the optic nerve; attenuated vessels; BLSP	Altered
US-4	No	II:1 (42)	USH1	Severe SNHL; prelingual	Altered	18 yo	27 yo	VF constriction (tunnel vision); pallor of the optic nerve; severe thinning of vessels; BLSP	Altered
US-5	No	II:5 (58)	USH1	Profound SNHL; prelingual	Altered	9 yo	21 yo	VF constriction (tunnel vision), right eye more affected; pallor of the optic nerve; thinning of the vessels; retinal and choroidal degeneration; BLSP in the posterior region	Abolished
US-6	Yes (1st cousins)	II:2 (43)	USH1	Severe SNHL; prelingual	Altered	7 yo	10 yo	Early onset RP; total amaurosis, no VF left; waxy pallor of the optic nerve; optic disc drusen; attenuated vessels; BLSP	Abolished
US-7	Not reported ^∗^1000–2000 population	II:1 (10)	USH1	Severe SNHL; prelingual; cochlear implant	Altered	7 yo	8 yo	VF and VA remain unaffected; mild thinning of the vessels; normal macula; fine colorless particles in the vitreous; few pigment accumulations	Altered
US-8	Yes (1st cousins)	II:1 (80)	USH?	Moderate SNHL; postlingual (adolescence); progressive in the last 10 years	Normal	10 yo	16 yo	VF constriction (tunnel vision); low VA; attenuated vessels; pallor of optic nerve; macular edema and cysts; choroidal vascular fibrosis; BLSP	Abolished
US-9	Yes (2nd cousins)	II:6 (61)	USH2	Mild SNHL; postlingual (adulthood); progressive	Normal	20 yo	30 yo	VF constriction (mid-peripheral ring scotoma); attenuated vessels; pallor of optic nerve; BLSP	Abolished
US-10	No	II:1 (59)	USH2	Moderate SNHL; postlingual (adolescence); non-progressive	Normal	19 yo	28 yo	VF constriction (tunnel vision); thinning and atrophy of	Abolished
US-11	Not reported ^∗^500–1000 population	II:1 (41)	USH1	Profound SNHL; prelingual	Altered	9 yo	10 yo	VF constriction (tunnel vision), right eye more affected; waxy pallor of the optic nerve; macular degeneration, BLSP	Altered
US-12	Yes (2nd cousins)	II:3 (47)	USH?	Severe SNHL; prelingual	Altered	18 yo	17 yo	VF constriction (tunnel vision), pallor of optic nerve; attenuated vessels; BLSP	Abolished
US-16	Yes, in the third generation (1st cousins)	II:5 (50)	USH2	Moderate SNHL; postlingual (adolescence); non-progressive	Normal	14 yo	26 yo	Severe VF reduction; severe thinning of vessels; pallor of optic nerve; BLSP	Abolished

*?, unclear USH category; SNHL, sensorineural hearing loss; NB, night blindness; yo, years old; Dx, diagnosis; VF, visual field; VA, visual acuity; ERG, electroretinogram. Asterisk indicates that the parents come from the same town with the specified number of inhabitants.*

The presence in homozygosis of p.Gln1221^∗^ in *PCDH15* led to a typical USH2 phenotype with a severe HL of postlingual onset, no vestibular dysfunction and late onset RP, and despite being the causative mutation a nonsense variant. Although it is not common, mutations in genes that usually lead to USH1 and cause a USH2 phenotype, and vice-versa, have been reported (Bonnet et al., 2011; Aparisi et al., 2014; Fuster-García et al., 2018).

The mutations c.1841-2A>G (p.Gly614Asp^∗^fs6) and c.2299delG (p.Glu767Serfs^∗^21) in *USH2A* have been reported many times in the literature as pathogenic in many populations.

Noteworthy, two mutations are recurrent in this study. The c.619C>T mutation (p.Arg207^∗^) in *CLRN1* was described by García-García et al. and Licastro et al. almost simultaneously in two *a priori* unrelated Spanish families of Basque origin and one family of Italian origin, respectively (García-García et al., 2012; Licastro et al., 2012). This mutation was found in homozygous state in two Cuban families. In the first family reported by García-García et al., the only affected member carried the p.Arg207^∗^ mutation together with p.Tyr63^∗^. The patient displayed bilateral severe progressive sensorineural HL corrected with hearing aids and was a candidate for cochlear implantation. She showed a delay in gait development and a vestibular hyporeflexia and she displayed typical symptoms of RP since young. The onset of her RP was at 9 years old, including night blindness and peripheral visual loss. Fundus ophthalmoscopy showed pigmentary anomalies typical of RP with a visual acuity of 0.4 in both eyes and a rapid progression of the visual loss.

In the second family there were two affected sibs who were compound heterozygotes for p.Arg207^∗^ and p.Ile168Asn. They displayed very discordant phenotypes. One brother had a typical RP and normal speech acquisition and motor milestones. At 13 years old he displayed a progressive bilateral HL that ranged 79–80 dB in the last clinical examination, and the vestibular function was normal. The other brother presented with a typical RP as well, but displayed a prelingual severe HL that required deaf school education.

These findings illustrate the impressive wide spectrum of sensorineural hearing impairment in type and degree, and the high degree of intersubject and intrafamiliar variability due to *CLRN1* mutations, as previously reported (Pennings et al., 2003).

The other mutation, c.4488G>C (p.Gln1496His) in *CDH23*, was described by Bolz et al. (2001) in a large Cuban family. That study allowed the identification of the *CDH23* gene as responsible of Usher syndrome type 1. Although c.4488G>C is a missense mutation (p.Gln1496His), the G>C change affects the last exon nucleotide and computational predictions and *in vitro* studies support the hypothesis of a splicing alteration leading to a truncated protein (Bolz et al., 2001).

Additionally, c.4488G>C has been reported two more times in the literature in two unrelated families of Spanish origin showing a typical USH1 phenotype (Astuto et al., 2002; Oshima et al., 2008).

It is noteworthy that the frequency of the mutated genes varies significantly when compared to other countries. In most populations *MYO7A* is the most prevalent gene among USH1 patients accounting for about 50% of the cases, except in some endogamic populations (Roux et al., 2011; Le Quesne Stabej et al., 2012; Glöckle et al., 2014; Yoshimura et al., 2014; Bonnet et al., 2016; Dad et al., 2016; Eandi et al., 2017; Sun et al., 2018). However, all the USH1 patients in this cohort carry mutations in *CDH23*. Furthermore, c.4488G>C accounts for 80% of USH1 alleles and no *MYO7A* mutations were detected in the cohort.

No conclusions can be obtained from the USH2 mutation distribution given the small size of the sample. Two out of the three clear USH2 patients are caused by mutations in *USH2A*, whereas the remaining is due to a mutation in *ADGRV1*. Both *USH2A* mutations have been reported many times in the literature, being c.2299delG the most frequent USH2 mutation in populations of European origin (Dreyer et al., 2000).

The frequency of Usher syndrome due to mutations in *CLRN1* in our sample is 18% (two out of 11), considerably higher than the 5% or less in other populations. Usher syndrome resulting from mutations in *CLRN1* is rare except in Finland and among the Ashkenazi jews, and its high frequency among USH3 patients in these populations is due to founder mutations (Joensuu et al., 2001; Ness et al., 2003). Here, the apparently high frequency of *CLRN1* is attributable to the presence of another unique mutation that probably has a Spanish origin.

It must be remarked that most of the mutations found in this study are homozygous, yet it could be possible that these were in fact heterozygous variants in concurrence of a large deletion, even when consanguinity is at stake. MLPA could be performed for mutations in *USH2A* and *PCDH15*, but there is no kit available to analyze the other implicated genes *ADGRV1*, *CLRN1*, and *CDH23*.

Segregation analysis would also help to unveil this issue and also to confirm if the compound heterozygous mutations are indeed in trans and, thus, causative of the disease. However, the obtainment of DNA samples of the relatives was not available.

Although the sample size is very small, it is tempting to speculate that the gene frequencies in Cuba are distinct from other populations, mainly due to an “island effect” and genetic drift. Further studies with a larger sample comprising different geographical regions of Cuba are needed to elucidate the real genetic landscape of Usher syndrome in the Cuban population.

## Ethics Statement

The institutional board of the Ethics Committee of the University Hospital La Fe and the University of Holguín, respectively, approved the study, according to the tenets of the Declaration of Helsinki and reviews.

## Author Contributions

JM and AL conceived, designed, and supervised the study. AL provided the samples. ES did the clinical data curation. CF-G, GG-G, and BG-B performed the molecular experiments and analyzed the sequencing data. EA and TJ did the results validations. JM and GG-G obtained the funding. ES and CF-G wrote the initial manuscript. JM, AL, and GG-G reviewed and edited the manuscript.

## Conflict of Interest Statement

The authors declare that the research was conducted in the absence of any commercial or financial relationships that could be construed as a potential conflict of interest. The reviewer CG declared a past co-authorship with several of the authors TJ and JM to the handling Editor.

## Footnotes

1 www.ampliseq.com

2 https://ionreporter.thermofisher.com/ir/

3 https://www.ncbi.nlm.nih.gov/SNP/

4 https://grenada.lumc.nl/LOVD2/Usher_montpellier/

5 https://www.emqn.org/emqn/Best+Practice
